# Gallstone Management in Patients Undergoing Laparoscopic Sleeve Gastrectomy

**DOI:** 10.1007/s11695-025-08048-4

**Published:** 2025-07-15

**Authors:** Tamer Abdelbaki

**Affiliations:** Alexandria University, Alexandria, Egypt

**Keywords:** Sleeve gastrectomy, Gallstone disease, Simultaneous cholecystectomy, Postoperative gallstone formation

## Abstract

**Background:**

The management of gallstones in patients undergoing bariatric surgery remains a topic of debate. This study assesses the management of gallstone disease (GSD) and the development of postoperative gallstones in patients undergoing laparoscopic sleeve gastrectomy (LSG).

**Methods:**

This study included all patients who underwent LSG at our institution between October 2020 and November 2023. Data on patient demographics, gallbladder status, operative details, complications, and outcomes were collected. Simultaneous cholecystectomy (SCC) was performed in patients with symptomatic GSD and in asymptomatic patients who agreed to undergo the procedure.

**Results:**

Among 2930 LSG patients, 391 (13.34%) had GSD, including 243 (8.29%) with gallstones and 148 (5.05%) with a history of cholecystectomy. SCC was performed in 142 (4.84%) patients, while 101 asymptomatic patients declined cholecystectomy. SCC increased operative time by 35 ± 11 min without an increase in the complications rate compared with LSG alone. Postoperative gallstones developed in 160 (6.3%) of primary gallstone-free patients and in five (4.95%) of the asymptomatic GSD patients who declined SCC. Postoperative cholecystectomy was performed after a mean of 6.11 ± 2.6 and 5.2 ± 2.4 months, respectively. Mean %EWL at cholecystectomy was 57.89 ± 14.83% and 49.81 ± 15.64%. Weight loss outcomes and improvements in obesity-related diseases were satisfactory.

**Conclusion:**

SCC is safe but results in a longer operative time with no increase in complication rates. Postoperative gallstone formation remains a concern, with more acute symptoms and earlier cholecystectomy at a lower %EWL in asymptomatic GS patients, compared to gallstone-free patients. Our findings showed that postoperative gallstones developed in only 4.95% of the asymptomatic GSD patients who declined SCC. Preoperative counseling is recommended to inform patients of the potential need for cholecystectomy following LSG.

## Introduction

The global rise in obesity represents a significant public health concern. Metabolic and bariatric surgeries (MBS) have gained prominence as effective treatment options for managing obesity, particularly in severe cases, offering sustainable outcomes and often leading to improvements or remission of obesity-associated conditions [1]. However, patients undergoing MBS are at an elevated risk of developing gallstones. This increased risk is attributed to a predisposition for cholesterol gallstone formation and the effects of altered neurohormonal regulation on gallbladder contractility in individuals with obesity. Furthermore, obesity itself is a known risk factor for gallbladder disease [2]. The rapid weight loss commonly associated with bariatric procedures further exacerbates this risk by increasing bile lithogenicity, thereby raising the likelihood of gallstone formation [3].

The management of the gallbladder in patients with obesity undergoing bariatric surgery remains a topic of ongoing debate, primarily due to the heightened risk of gallstone formation and the controversy surrounding prophylactic cholecystectomy. Advocates of prophylactic cholecystectomy argue that the combination of obesity and the rapid weight loss following bariatric surgery significantly increases the likelihood of gallstone formation. They contend that preemptive gallbladder removal can prevent complications, reduce the need for emergency surgeries, and potentially be more cost-effective in the long term [4].

Conversely, critics argue that routine cholecystectomy is unnecessary for many patients, as not all develop symptomatic gallstones, and it may expose them to avoidable surgical risks. An alternative approach, endorsed by some experts, is simultaneous cholecystectomy (SCC), particularly when preoperative ultrasonography detects gallbladder pathology [5]. Others recommend limiting SCC to patients with symptomatic gallstones, thereby optimizing the balance between surgical risks and benefits [6].

The relationship between %EWL, early gallstone formation, and the postoperative presentation of symptomatic and asymptomatic gallstones requires further evaluation. This manuscript examines the management of gallstone disease (GSD), the incidence of postoperative gallstone formation, and the subsequent need for cholecystectomy in patients undergoing laparoscopic sleeve gastrectomy (LSG) at a tertiary care center in Egypt.

## Patients and Methods

This study included all patients who underwent metabolic and bariatric surgery (MBS) at our institution over a three-year period, from October 2020 to November 2023. Demographic and anthropometric data were collected, along with information on obesity-associated medical conditions such as type 2 diabetes mellitus (T2DM), hypertension, and obstructive sleep apnea. As part of the preoperative evaluation, routine gallbladder ultrasound was performed, with magnetic resonance cholangiopancreatography (MRCP) conducted when indicated. The presence and type of gallbladder disease (GBD, as well as the occurrence and duration of biliary symptoms, were carefully documented. Patients with a history of prior MBS, previous cholecystectomy (CC), and/or management of common bile duct (CBD) stones before undergoing bariatric surgery were excluded from the study.

Patients with symptomatic gallstones (GS) were scheduled for simultaneous cholecystectomy (SCC) during LSG whenever feasible. For patients with asymptomatic GS identified on preoperative ultrasonography, SCC was offered as an option, accompanied by detailed counseling. Patients with a normal GB or asymptomatic GS who opted against SCC proceeded with LSG alone and were prescribed postoperative prophylactic ursodeoxycholic acid (UDCA) to mitigate the risk of GS formation. During postoperative follow-up, patients who developed GBD-related symptoms after LSG were managed with subsequent laparoscopic cholecystectomy (LC).

The study population was evaluated through a comprehensive assessment of preoperative gallbladder status, operative time for both LSG and SCC, complication rates, incidence of new postoperative gallstones, and the need for subsequent cholecystectomy. Additionally, the study analyzed the interval between LSG and cholecystectomy, as well as postoperative weight loss, measured by the percentage of excess weight loss (%EWL) and the percentage of excess body mass index loss (%EBMIL).

Patients were monitored at the bariatric surgery clinic for at least one year, with gallbladder ultrasonography conducted at 3, 6, and 12 months postoperatively. The study was approved by the Institutional Review Board (IRB), (IRB number: 012098) and conducted in accordance with the principles of the Helsinki Declaration. Verbal consent was obtained from all participants prior to their inclusion in the study.

## Statistical Analysis

Data were analyzed using IBM SPSS software package, version 18 (IBM Company, Chicago, IL, USA). Results were reported as mean ± standard deviation for continuous variables or as percentages for categorical variables. Comparison of means for continuous variables was done using *t* tests while the comparison of categorical variables was done using chi-squared tests or Fisher’s exact *t* test when appropriate. A *p* value of < 0.05 was considered significant.

## Results

**Fig. 1:**
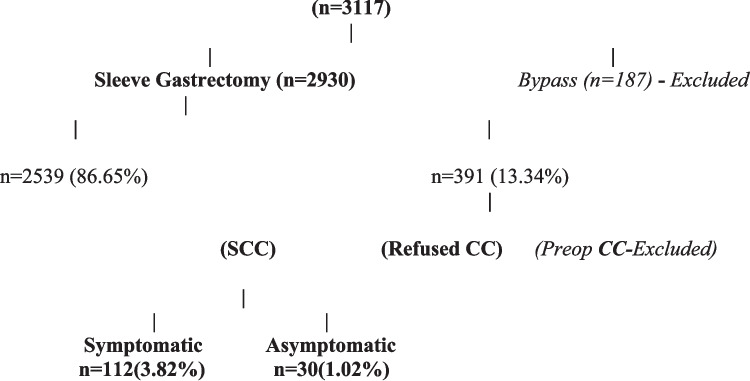
Gall stones in patients undergoing LSG

During the study period, 3117 patients admitted to our surgery unit were eligible for metabolic and bariatric surgery (MBS). Among them, 187 underwent gastric bypass procedures, including One-Anastomosis Gastric Bypass (OAGB) and Roux-en-Y Gastric Bypass (RYGB), while 2930 underwent laparoscopic sleeve gastrectomy (LSG). This study included only patients who underwent LSG, excluding those who underwent gastric bypass due to their relatively small sample size. Notably, of the 2930 LSG patients, 391 (13.34%) had either gallstones (GS) [243 patients (8.29%)] or a history of cholecystectomy for GS [148 patients (5.05%)] (Fig. 1).

Patients were classified into four groups based on preoperative clinical assessments and gallbladder ultrasonography. Group A comprised 2539 patients (86.66%) with a normal gallbladder (GB) who underwent LSG alone. Group B included 142 patients (4.84%) who underwent simultaneous cholecystectomy (SCC) during LSG. This group consisted of 112 patients (3.82%) with symptomatic GB pathology (gallstones or biliary sludge) and 30 patients (1.02%) with asymptomatic cholelithiasis who opted for SCC after preoperative counseling. Group C comprised 101 patients (3.44%) with asymptomatic cholelithiasis who declined SCC and underwent only LSG. Additionally, 148 patients (5.05%) had previously undergone CC (Group D) and were excluded from the study.

Table 1 provides the baseline characteristics and prevalence of obesity-associated diseases for all study participants. No significant differences were observed between the groups in terms of mean age, sex distribution, preoperative weight, BMI, or the presence of associated medical conditions. The mean age was 39 ± 10.26 years, and the mean BMI was 47.32 ± 6.28 kg/m^2^. Additionally, 2507 (90.11%) of the patients were female.

**Table 1:** Baseline characteristics and prevalence of obesity-associated diseases

	Group A	Group B	*p*	Group C	*p*	*Total*
	(*n* = 2539)	(*n* = 142)		(*n* = 101)		(*n* = 2782)
**Sex female**						
*n*(%)	**2297(90.4%)**	** 139(97.2%)**	*0.0758*	**93(92.07%)**	*0.068*	**2507(90.11%)**
**Age (years)**						
Mean ± SD	39.1 ± 11.4	40 ± 10.5	*0.0681*	42.5 ± 10.7	*0.072*	39 ± 10.26
(Range)	(18.0–67.0)	(22–66)		(28–62)		(18–67)
**Weight (kg)**						
Mean ± SD	**113 ± 20**	**114 ± 22**	*0.0597*	**117 ± 24**	*0.068*	**140 ± 20.64**
(Range)	(78–216)	(80–210)		(75–197)		(75–216)
**BMI (kg/m**^**2**^**)**						
Mean ± SD	**41.05 ± 6.24 **	**42.07 ± 6.48**	*0.0693*	**43.40 ± 6.7**	*0.125*	**47.32 ± 6.28**
(Range).	(30.2–67.0)	(30.5–64.8)		(32.1–60.8)		(30.2–67.0)
**Associated diseases: *n*(%)**						
T2DM	349 (13.74%)	18 (12.67%)	*0.814*	13 (12.87%)	*1.0*	380(13.69%)
Hypertension	285 (11.22%)	17 (11.97%)	*0.891*	12 (11.88%)	*1.0*	314(11.28%)
OSA	41 (1.61%)	3 (2.11%)	*0.908*	5 (4.95%)	*0.391*	49(1.76%)
Joint pain	50 (1.96%)	2 (1.40%)	*0.874*	2 (1.98%)	*1.0*	54(1.94%)
PCOS	18 (0.70%)	1 (0.70%)	*1.0*	2 (1.98%)	*0.765*	21(0.75%)
Asthma	131(5.15%)	12(8.45%)	*0.137*	10(9.90%)	*0.872*	153(5.49%)

*BMI* body mass index, *kg* kilogram, *n* number, *T2DM* diabetes mellitus,*OSA* obstructed sleep apnea, *PCOS* polycystic ovary syndrome, *CJP* chronic joint pain.(Data expressed as a percentage (%) or mean ± standard deviation), *p =  < 0.05 (significant)

The mean operative time, length of hospital stay, and surgical complications are summarized in Table 2. The mean operative duration was 61.12 ± 4.75 min (range: 50–75 min) for Group A, 63.13 ± 7.62 min (range: 50–80 min) for Group C, and 94.19 ± 8.46 min (range: 70–110 min) for Group B. In Group B, the addition of SCC extended the operative time by an average of 35 ± 11 min (range: 30–45 min). Technical difficulties related to excessive intra-abdominal fat and significantly enlarged liver were encountered in only two SCC patients, with no impact on surgical outcomes. There were no significant differences in the length of hospital stay between the groups.

**Table 2:** Mean operative time, hospital stay, and complications

	Group A	Group B	*p*	Group C	*p*
	(*n* = 2539)	(*n* = 142)		(*n* = 101)	
Mean operative time (min)	61.12 ± 4.75	94.19 ± 8.46^*^	< *0.0001*	63.13 ± 7.62^*^	< *0.0001*
(Range)	(50–75)	(70–110)		(50–80)	
Mean hospital stay (days)	1.21 ± 0.39	1.17 ± 0.39	*0.236*	1.20 ± 0.23	*0.453*
	(1–2)	(1–2)		(1–2)	
Complications *n* (%)	19 (0.75%)	3 (2.11%)	0.063 2	(1.98%)	*1.0*
Bleeding *n* (%)	7 (0.27%)	1 (0.70%)		0 (0.00%)	
Biliary injury *n* (%)	0 (0.00%)	0 (0.00%)		0 (0.00%)	
Gastric leak *n* (%)	0 (0.00%)	0 (0.00%)		0 (0.00%)	
Port site infection	12 (0.47%)	2 (1.45%)		1 (0.99%)	
Pneumonia	0 (0.00%)	0 (0.00%)		1 (0.99%)	
Pulmonary embolism	0 (0.00%)	0 (0.00%)		0 (0.00%)	

A = no gallstones (sleeve gastrectomy only), B = simultaneous cholecystectomy, C = asymptomatic gallstones (sleeve gastrectomy only), *p =  < 0.05 (significant).[Data expressed as a percentage (%) and mean ± standard deviation (SD)]

No mortality or major complications were reported in this study. Operative complications, including bleeding, port-site infection, and pneumonia, are summarized in Table 2. The mean follow-up period for the study participants was 18 ± 2 months (range: 6–36 months). Patients attended follow-up visits at 3, 6, and 12 months. The follow-up rate at 3 and 6 months varied between 76.4% and 89.8%, respectively, while 92.77% of patients completed the 12-month follow-up visit.

The incidence of postoperative gallstone (GS) formation, biliary symptoms, subsequent cholecystectomy (CC), and weight loss outcomes following LSG are summarized in Table 3. In Group A, 160 patients (6.3%) developed gallstone disease (GSD), with 2.08% experiencing acute cholecystitis. Among those who developed postoperative GS, 70 required CC, which was performed at a mean interval of 6.11 ± 2.6 months (range: 3–18 months) after LSG. In Group C, five patients (4.95%) developed acute biliary symptoms and subsequently underwent CC after a mean postoperative interval of 5.2 ± 2.4 months (range: 2–8 months).

**Table 3:** Postoperative gallbladder disease, and weight loss progress

	Group A	Group C	*p*	Total
	(*n* = 2539)	*(n = 101)*		* (n = 2640)*
**Postoperative GS/symptoms**				
* n* (%)	*160 (6.3%)*	* 5 (4.95%) *	*0.833 *	*165 (6.25%)*
GS symptoms *n* (%)				
Biliary colic	75 (2.95%)	1 (0.99%)		76 (3.94%)
Acute cholecystitis	53 (2.08%)	4 (3.96%)		57 (6.04%)
Asymptomatic (US)	32 (1.26%)	0 (0.00%)		32 (1.26%)
Pancreatitis	0 (0.00%)	0 (0.00%)		0 (0.00%)
**Subsequent CC**	70 (2.75%)	5 (4.95%)	*0.208*	75 (2.84%)
**Mean time to CC (m)**	6.11 ± 2.6	5.2 ± 2.4^*^	< *0.001*	
(Range)	(3–18)	(2–8)		
**% EWL** **at CC **	57.89 ± 14.83	49.81 ± 15.64^*^	< *0.001*	
**% EBMIL at CC **	68.54 ± 16.53	58.38 ± 22.16^*^	< *0.001*	

*GS* gallstones, *n* number, *US* ultrasound finding, *CC* cholecystectomy, *m* months,*EWL* excess weight loss, *EBMIL* excess BMI loss

*p =  < 0.05 (significant)

Patients who developed postoperative gallstones had a higher %EWL at 12 months compared to those who did not, with mean values of 71.59% ± 8.68 (range: 49.69–96.05) and 69.13% ± 12.83 (range: 24.04–143.20), respectively. Postoperative weight loss progression and the improvement of obesity-associated conditions are summarized in Table 4. Patients who completed 12 months of follow-up achieved significant weight loss, with a mean %EWL of 75.11% ± 12.74 in Group A, 72.57% ± 3.60 in Group B, and 73.21% ± 6.72 in Group C. However, at the time of postoperative cholecystectomy, the mean %EWL was significantly lower in Group C patients compared to Group A patients: 49.81% ± 15.64 and 57.89% ± 14.83, respectively (Table 3).

**Table 4:** Weight loss and resolution or improvement of associated diseases

	Group A (*n* = 2539)	Group B (*n* = 142)	*p* value	Group C (*n* = 101)	*p* value
**%EWL**					
3 m	26.56 ± 5.27%	25.1 ± 3.43%	0.071	26.31 ± 2.42%	0.069
6 m	55.51 ± 9.51%	52.85 ± 4.02%	0.060	55.37 ± 2.82%	0.062
12 m	75.11 ± 12.74%	72.57 ± 3.60%	0.063	73.21 ± 6.72%	0.070
**%EBMIL**					
3 m	31.63 ± 6.60%	30.21 ± 5.13%	0.080	31.02 ± 3.64%	0.089
6 m	66.21 ± 12.74%	63.54 ± 7.79%	0.063	65.24 ± 5.29%	0.078
12 m	89.71 ± 17.18%	87.26 ± 10.06%	0.071	86.25 ± 9.60%	0.082
**T2DM**					
	*n* = 349(13.74%)	*n* = 18(12.67%)		*n* = 13(12.87%)	
3 m	209(59.88%)	11(61.11%)		9(69.23%)	0.9438
6 m	302(86.53%)	15(83.33%)		11(84.61%)	0.9564
12 m	316(90.54%)	17(94.44%)		12(92.30%)	0.9553
**Hypertension**					
	*n* = 285(11.22%)	*n* = 17(11.97%)		*n* = 12(11.88%)	
3 m	116(40.70%)	5(29.41%)		4(33.33%)	0.9547
6 m	160(56.14%)	9(52.94%)		7(58.3%)	0.6951
12 m	210(73.68%)	12(70.58%)		9(75.00%)	0.9991
OSA	*n* = 41(1.61%)	*n* = 3(2.11%)		*n* = 5(4.95%)	
12 m	*n* = 41(100%)	*n* = 3(100%)		*n* = 5(100%)	1.0000

*%EWL,* percent excess weight loss; *%EBMIL,* percentage excess body mass index loss; *T2DM,* diabetes mellitus; *OSA,* obstructed sleep apnea, *p* =  < 0.05 (significant)

## Discussion

Obesity is a significant risk factor for gallstone disease (GSD), with prevalence rates ranging from 22.8% to 43.6% among individuals undergoing metabolic bariatric surgery (MBS) [7]. In this study, 13.34% of the 2930 patients eligible for laparoscopic sleeve gastrectomy (LSG) were identified as having either gallstones (8.29%) or a history of cholecystectomy (CC) due to GSD (5.05%). Patients with a history of preoperative CC were not included in this study.

Assessing gallbladder condition and identifying potential procedural challenges during MBS can help improve risk-adjusted outcomes. The management of MBS patients with gallstones (GS) and the optimal timing of cholecystectomy remain subjects of ongoing debate. Current approaches include performing simultaneous cholecystectomy (SCC) for all GS patients, limiting CC to symptomatic cases, or conducting prophylactic CC in all instances.

In the present study, SCC during LSG was performed in 142 (4.84%) of all eligible patients, including all symptomatic patients and those with asymptomatic GS who consented to the procedure (Group B). Patients with asymptomatic GS who declined SCC (Group C) and preoperatively gallstone-free patients (Group A), underwent LSG alone. There was no significant difference in baseline characteristics and the prevalence of obesity-associated diseases among the studied groups. Previously reported incidences of SCC during MBS are approximately 2% [8, 9]. An analysis by Wood et al. of the 2015 MBSAQIP registry indicated that 2% of 98,292 LSG patients underwent SCC [10]. Advocates of SCC for patients with gallbladder pathology argue that it is a safe and effective strategy that reduces the risk of future complications and the need for reoperation [5]. However, others recommend restricting laparoscopic cholecystectomy (LC) to symptomatic patients, emphasizing the low incidence (< 5%) of subsequent cholecystitis or symptom development in asymptomatic cases [11, 12]. For minimally symptomatic patients, delayed or selective LC is considered acceptable, given the consistently low long-term morbidity previously reported [6].

Conversely, some experts discourage simultaneous interventions during bariatric procedures due to technical challenges, including excessive intra-abdominal fat, difficulty establishing pneumoperitoneum, and differing port placements [13]. Additionally, the higher incidence of bile duct injuries further complicates this approach [14]. Elgohary et al. recommend delaying cholecystectomy by approximately two months post-bariatric surgery to benefit from reduced BMI, less intra-abdominal fat, smaller liver size, and fewer adhesions in Calot’s triangle, minimizing biliary complications [7]. Similarly, Morais et al. found that delaying cholecystectomy simplifies the procedure [15]. This strategy also allows for managing late bariatric surgery complications [7, 16]. Ultimately, some experts advocate a balanced approach, weighing the risks of SCC against the morbidity of delayed, gallstone-related complications [17]. However, the necessity of prophylactic SCC during sleeve gastrectomy (SG) remains debated due to the high incidence of postoperative biliary complications [4, 18].

In Group B, patients who underwent SCC had a mean operative time of 94.19 ± 8.46 min (range: 70–110), with the cholecystectomy (CC) specifically accounting for a median of 35 ± 11 min (range: 30–45). In contrast, mean operative times in Groups A and C were significantly less, at 61.12 ± 4.75 min (range: 50–75) and 63.13 ± 7.62 min (range: 50–80), respectively. Hospital stays did not differ significantly among the groups. The additional time required for SCC during MBS varied depending on technical challenges. Consistent with our findings, Dakour-Aridi et al. reported an extra 33 min for CC [9], while Wood et al. noted increases of 27 min for LSG and 28 min for OAGB/MGB [10]. Raziel et al. observed a 35-min increase in SCC for symptomatic GS patients without prolonged length of stay (LOS) or higher complication rates [19]. Dincer et al. reported an operative time increase of 18–49 min [20], while Coskun et al. found SCC added 49.1 ± 27.9 min without significant complications [7, 21].

Previous studies have reported higher complication rates in laparoscopic sleeve gastrectomy (LSG) with simultaneous cholecystectomy (SCC), often attributed to severe obesity, an enlarged liver, and limited visualization of Calot’s triangle [11, 22]. However, our study found no major complications or mortality, and postoperative complication rates were not statistically significant between groups (*p* > 0.05). Among the 142 SCC patients, 2.11% experienced complications, none of which were directly related to cholecystectomy. No bile leaks or bile duct injuries were reported. However, one SCC patient (0.70%) developed a common bile duct stone 8 months postoperatively, which was successfully treated with endoscopic retrograde cholangio-pancreatography (ERCP) followed by a subsequent cholecystectomy.

Intraoperative bleeding rates were 0.27%, 0.7%, and 0.0% in Groups A, B, and C, respectively, with all cases successfully managed intra-operatively without the need for re-intervention. No postoperative bleeding was observed.

Port site infections (PSIs) occurred at rates of 0.47%, 1.45%, and 0.99% in Groups A, B, and C, respectively. Wood et al. found that simultaneous cholecystectomy (SCC) during laparoscopic sleeve gastrectomy (LSG) increased the risk of surgical site infection (SSI) by 0.6%, from 0.4% to 1.0% [10]. Similarly, Elgohary et al. reported a 2.6% PSI rate in LSG patients who underwent SCC, compared to 0.0% in those who did not (p < 0.001), with an overall adverse event rate of 15.8% in SCC patients versus 8.3% in non-SCC patients [7].

In contrast, Tustumi et al. reported that SCC reduced postoperative complications and reoperations compared to performing cholecystectomy separately after bariatric surgery [22]. Similarly, Juo et al. found no significant difference in complication rates between SCC and interval cholecystectomy [11, 23]. Dakour-Aridi et al., in a study of 21,137 LSG patients (2% of whom underwent SCC), reported no significant increase in overall adverse events, though SCC patients had slightly higher rates of bleeding and pneumonia compared to those undergoing LSG alone (5.7% vs. 4.7%) [9]. Yardimici et al. also found no increase in operative complications among LSG patients with gallstones, whether symptomatic or asymptomatic. Notably, 79.2% of asymptomatic gallstone patients remained symptom-free despite significant weight loss [16].

Among 2539 gallstone-free LSG patients (Group A), 160 (6.3%) developed postoperative gallstones (GS) after a mean duration of 6.11 ± 2.6 months. Biliary colic was the most common presentation (2.95%), followed by acute cholecystitis (2.08%). Additionally, asymptomatic GS was incidentally detected in 1.26% of patients during routine postoperative ultrasound. Postoperative cholecystectomy was performed in 70 patients (2.75%).

The incidence of symptomatic gallstone disease (GSD) after metabolic and bariatric surgery (MBS) ranges from 7 to 15% [24]. Dirnberger et al. reported a 9.3% incidence of de novo symptomatic gallbladder disease (GBD) in initially gallstone-free MBS patients, with rates of 7% following laparoscopic Roux-en-Y gastric bypass (LRYGB) and 2.3% after LSG. Biliary colic was the most common presentation; however, 19.8% of symptomatic patients initially presented with complications such as cholecystitis, biliary pancreatitis, or choledocholithiasis [11]. Similarly, Nagem et al. reported a 28.9% incidence of GS within a median follow-up of three years, with 15.7% of cases becoming symptomatic. While biliary colic was the predominant presentation, some patients developed severe complications, including acute cholecystitis, cholangitis, choledocholithiasis, and pancreatitis [25]. Li et al. also observed complicated GBD in 1.9% of initially gallstone-free patients following bariatric surgery [26]. The rate of postoperative GS formation varies depending on the bariatric procedure. Some studies have reported a higher incidence following LRYGB compared to LSG [11, 27], while others have found no significant difference [28].

Of the 101 Group C patients with asymptomatic gallstones (GS) who underwent LSG alone, five (4.95%) developed acute biliary symptoms, and all required cholecystectomy after a mean duration of 5.2 ± 2.4 months. Morais et al. reported that 3.3% of patients with asymptomatic gallbladder (GB) pathology developed symptomatic GS within the first year following bariatric surgery [15]. Studies have documented the postoperative progression from asymptomatic to symptomatic gallbladder disease (GBD), with reported incidences ranging from 5% [29] to 9.3%–17.6% [19, 29]. Raziel et al. found that 9.3% (4 of 43) of patients required laparoscopic cholecystectomy (LC) after LSG [19], while Sioka et al. observed a 13% rate (3 of 23) [30]. Elgohary et al. reported that 33% of initially asymptomatic GS cases became symptomatic, with the proportion of symptomatic cases increasing from 25% preoperatively to 50% within 2 months post-MBS [7]. Additionally, El Hadidi et al. identified a family history of gallstone disease (GSD), higher preoperative BMI, and increased percentage of excess weight loss (%EWL) as independent risk factors for postoperative symptomatic cholelithiasis [31].

In this study, post-LSG cholecystectomy (CC) was performed in 2.75% of gallstone-free patients and 4.95% of those with asymptomatic gallstones (GS). The incidence of CC after metabolic and bariatric surgery (MBS) remains a topic of debate, with reported rates ranging from 2.9% to 10.6% [24, 32]. Altieri et al. documented CC rates of 6.5%, 9.7%, and 10.1% following laparoscopic adjustable gastric banding (LAGB), laparoscopic Roux-en-Y gastric bypass (LRYGB), and laparoscopic sleeve gastrectomy (LSG), respectively, underscoring the need for preoperative counseling regarding CC risk [24]. Moon et al. reported a 6.1% incidence of postoperative CC after LSG when SCC was performed for all patients with a positive preoperative ultrasound [28]. In contrast, Dinberger et al. reported a 7.8% incidence when SCC was limited to symptomatic patients [11]. Keilani et al., in an analysis of 1751 MBS patients, found that 4.2% required postoperative CC, with most cases occurring within 6 months [33]. Additionally, a cohort study reported CC rates of 9.3% in patients with asymptomatic GS and 2.7% in those without GS within the first year [19].

In the present work, the mean duration between LSG and CC was significantly shorter in Group C patients with asymptomatic gallstones compared to preoperatively gallstone-free Group A patients: 5.2 ± 2.4 months (range 2–8) versus 6.11 ± 2.6 months (range 3–18), respectively. The median interval between bariatric surgery and CC has been reported to range from 8 to 18 months [11]. Hakim et al. found that 50% of patients underwent CC within 2–10 months post-LSG, 17.5% between 10–15 months, and 32.5% after 16 months [8].

This study demonstrated that patients who developed GS (6.25%) had a significantly higher mean %EWL in the first postoperative year compared to those who did not (71.59 ± 8.68 vs. 69.13 ± 12.83). However, our results showed that at the time of CC, the mean %EWL was significantly lower in Group C patients with asymptomatic GS than in gallstone-free Group A patients (49.81 ± 15.64% vs. 57.89 ± 14.83%, respectively). Hakim et al. reported that at the onset of GBD, 17.5% of patients had 21%–25% EWL, 40% had 26%–35% EWL, and 27% had EWL exceeding 36% [8]. Notably, weight loss greater than 25% EWL, particularly within the first 10 months post-LSG, was associated with a higher incidence of GBD [8]. Similarly, Morais et al. found that symptomatic GS patients tended to achieve higher %EWL [15].

The mean percentage of excess weight loss (%EWL) and the improvement of obesity-associated diseases were satisfactory and consistent with previous LSG outcomes [34]. Patients who completed 12 months of follow-up also demonstrated successful weight loss. In our study, most obesity-associated diseases improved or resolved within 6 months. T2DM remission began in the first postoperative month, reaching 86.53%, 83.33%, and 84.61% in Groups A, B, and C, respectively, by 6 months. These findings align with previous studies reporting remission rates of 26%–80% at various intervals [35]. Hypertension showed significant improvement, consistent with the findings of Xiaoqiang et al., who observed a reduction in blood pressure within 10 days postoperatively and its resolution in 87% of patients by 12 months [36]. This suggests that LSG lowers blood pressure before substantial weight loss, likely due to neural and hormonal mechanisms. Additionally, the improvement in chronic joint pain and sleep apnea in our patients aligns with the findings of Xiaoqiang et al., who reported joint pain resolution in 78% of patients and sleep apnea resolution in 86% within 12 months [36].

## Limitations

The main limitations of this study include the relatively small number of patients with symptomatic or asymptomatic gallstones who underwent simultaneous cholecystectomy (SCC). Additionally, long-term follow-up of asymptomatic patients who declined SCC was not conducted. A multi-center study may help address these limitations.

## Conclusion

Simultaneous cholecystectomy (SCC) is a safe procedure that results in a longer operative time but does not significantly increase complication rates compared to laparoscopic sleeve gastrectomy (LSG) alone. Postoperative gallstone formation occurred in 6.3% of patients who were gallstone-free preoperatively, while postoperative symptoms developed in only 4.95% of those with preexisting asymptomatic gallstones who declined SCC. Postoperative symptoms were more acute, occurred after a shorter interval, and developed at a lower %EWL in asymptomatic GS patients compared to gallstone-free patients. Preoperative counseling is recommended to inform patients with asymptomatic gallstones about the potential need for cholecystectomy following LSG.

## Data Availability

No datasets were generated or analysed during the current study.
